# Identification and characterization of endogenous retroviruses upon SARS-CoV-2 infection

**DOI:** 10.3389/fimmu.2024.1294020

**Published:** 2024-04-05

**Authors:** Xuefei Guo, Yang Zhao, Fuping You

**Affiliations:** Institute of Systems Biomedicine, Department of Immunology, School of Basic Medical Sciences, Beijing Key Laboratory of Tumor Systems Biology, National Health Commission (NHC) Key Laboratory of Medical Immunology, Peking University Health Science Center, Beijing, China

**Keywords:** COVID-19, SARS-CoV-2, TEs, ERVs, Erv1, RNA-Seq

## Abstract

Endogenous retroviruses (ERVs) derived from the long terminal repeat (LTR) family of transposons constitute a significant portion of the mammalian genome, with origins tracing back to ancient viral infections. Despite comprising approximately 8% of the human genome, the specific role of ERVs in the pathogenesis of COVID-19 remains unclear. In this study, we conducted a genome-wide identification of ERVs in human peripheral blood mononuclear cells (hPBMCs) and primary lung epithelial cells from monkeys and mice, both infected and uninfected with SARS-CoV-2. We identified 405, 283, and 206 significantly up-regulated transposable elements (TEs) in hPBMCs, monkeys, and mice, respectively. This included 254, 119, 68, and 28 ERVs found in hPBMCs from severe and mild COVID-19 patients, monkeys, and transgenic mice expressing the human ACE2 receptor (hACE2) and infected with SARS-CoV-2. Furthermore, analysis using the Genomic Regions Enrichment of Annotations Tool (GREAT) revealed certain parental genomic sequences of these up-regulated ERVs in COVID-19 patients may be involved in various biological processes, including histone modification and viral replication. Of particular interest, we identified 210 ERVs specifically up-regulated in the severe COVID-19 group. The genes associated with these differentially expressed ERVs were enriched in processes such as immune response activation and histone modification. HERV1_I-int: ERV1:LTR and LTR7Y: ERV1:LTR were highlighted as potential biomarkers for evaluating the severity of COVID-19. Additionally, validation of our findings using RT-qPCR in Bone Marrow-Derived Macrophages (BMDMs) from mice infected by HSV-1 and VSV provided further support to our results. This study offers insights into the expression patterns and potential roles of ERVs following viral infection, providing a valuable resource for future studies on ERVs and their interaction with SARS-CoV-2.

## Introduction

Coronavirus disease 2019 (COVID-19), caused by severe acute respiratory syndrome coronavirus 2 (SARS-CoV-2), has emerged as a significant global health threat, infecting millions worldwide and resulting in numerous fatalities (1–3). Understanding the molecular events occurring during SARS-CoV-2 infection is imperative to control virus spread and effectively treat severe cases. Upon viral infection, host cells activated defensive responses to combat the invading pathogens (4–6). Utilizing next-generation sequencing (NGS) technology, numerous studies have investigated the gene expression patterns of host cells, thereby advancing our understanding of virus–host interactions (7–9). However, most of these studies have focused on profiling the expression of coding genes within the host, neglecting the exploration of transposable elements (TEs) and their expression patterns and functions.

Notably, TEs constitute nearly 50% of the human genome, whereas coding genes make up only 2% (10, 11). TEs encompass various elements such as endogenous retroviruses (ERVs), which are part of the long terminal repeat (LTR) family, long interspersed nuclear elements (LINEs), short interspersed nuclear elements (SINEs), and DNA transposons. Over millions of years, TEs have played a significant role in shaping the size and structure of our genome due to their transposition abilities (12–14). Many TEs have undergone mutations leading to loss of function during evolution (15). Consequently, numerous TEs may no longer generate the necessary proteins for complete transposition (16). Remarkably, some TEs capable of retrotransposition could insert sequences into new locations within the host genome. If integrated into or near coding gene exons, these elements may exert unforeseen effects on host gene regulation (17, 18). Consequently, abnormal TE expression may contribute to the pathogenesis of various diseases, including infectious diseases, autoimmune disorders, and cancers (19). On the contrary, the host organism could leverage TE sequences to facilitate specific biological processes—for instance, in mammals, genes derived from the ERV envelope protein could produce syncytin, a protein that promotes cell–cell fusion (12, 20). Additionally, the host can employ ERV sequences as enhancers to regulate gene expression during placental development. More recently, research has demonstrated that certain TE sequences can participate in the regulatory network of the host, modulating the expression of coding genes involved in the innate immune response following treatment with interferon γ (21–23).

Recent studies have highlighted a potential association between SARS-CoV-2 infection and ERVs—for example, researchers have shown that SARS-CoV-2 can induce the expression of the envelope protein of HERV-W in COVID-19 patients compared to healthy individuals (24–27). Furthermore, the overexpression of HERV-R induced by SARS-CoV-2 has been found to inhibit viral replication through ERK-mediated mechanisms (28). ERVs may play a role in the pathogenesis of COVID-19, as HERV-K113-ENV has been identified as a biomarker for assessing the severity of SARS-CoV-2 infection (29). In addition, Nicole et al. conducted the first comprehensive analysis of HERV loci expression in peripheral blood mononuclear cells (hPBMCs) during SARS-CoV-2 infection, revealing a dynamic modulation across COVID-19 convalescent stages (six samples) and individuals retesting positive after recovery (10 samples) (30). However, there is still a gap in research focusing on distinguishing TEs and ERVs among healthy donors, as well as mild and severe COVID-19 patients, at the whole-genome scale.

In this study, we aimed to investigate the genome-wide expression patterns and potential functions of ERVs following SARS-CoV-2 infection. We analyzed a comprehensive dataset comprising 99 bulk RNA-seq samples from human, monkey, and transgenic mice expressing the human ACE2 receptor (hACE2), sourced from the Gene Expression Omnibus (GEO) database. Additionally, we conducted independent RNA-seq experiments using mouse bone marrow-derived macrophages (BMDMs) infected with vesicular stomatitis virus (VSV) and herpes simplex virus 1 (HSV-1). These experiments allowed us to assess the reliability and accuracy of our pipeline in identifying TEs and exploring specific ERV subfamilies and loci affected by viral infection.

## Materials and methods

### Sample collections

We acquired 99 samples from the GEO database, including datasets GSE152418,GSE147507, GSE158297, GSE150728, and CRA002390. These samples comprised 52 human PBMCs (hPBMCs), 24 primary human bronchial epithelial cells (HBECs), 16 monkey lung epithelial cells, and seven mouse lung epithelial cells bulk RNA-seq samples with or without SARS-CoV-2 infection. Each sample had at least three biological replicates under various treatments or conditions, with detailed information provided in **Supplementary Table 1**. Specifically, dataset GSE152418, consisting of hPBMCs, contained 17 COVID-19 patient samples and 17 healthy control samples. These samples were categorized into different groups based on disease severity, such as ICU, severe, moderate, convalescent, and healthy. Our analysis consolidated the four ICU samples into the severe group and eliminated the two convalescent samples, as outlined in **Supplementary Table 2**.

### RNA-seq data analysis

The RNA-seq datasets utilized in this study were sourced from the GEO repository, encompassing GSE152418 for hPBMCs, GSE147507 for HBECs, GSE158297 for monkeys and mice, and GSE150728 along with CRA002390 to validate the hPBMC results. FastQC and Trim-Galore software were employed to ensure the quality control of the raw data and to generate clean data. Subsequently, the clean data was aligned to their respective reference genomes, such as hg19, rheMac10, and mm10, using Subread software (31). The Counts feature from Subread was then utilized to derive the gene expression matrix (32). Subsequently, the FPKM formula was applied to normalize the coding gene expression matrix. Differential expression analysis was conducted using the R package DESeq2 (33).

### GO annotation and KEGG pathway enrichment analysis

In the present study, the differentially expressed genes (DEGs) were subjected to enrichment analysis through Gene Ontology (GO) annotation and Kyoto Encyclopedia of Genes and Genomes (KEGG) pathway analysis. This was conducted using the clusterProfiler R package, specifically employing the enrichGO and enrichKEGG functions (34) or, alternatively, utilizing the online database DAVID (35). Significance thresholds were set such that GO and KEGG terms with false discovery rates (FDR) less than 0.01 were considered indicative of meaningful enrichments.

### TE identification

In our analysis, the software TEtranscripts (36) was employed to assess the abundance and expression of TE subfamilies. TEtranscripts was specifically designed for quantifying TE subfamily expression by associating multi-mapped reads with TE subfamilies. The TE annotation GTF files for humans, monkeys, and mice were derived from their respective DFAM repeat annotation files. TEtranscripts was executed with the following parameters: TEtranscripts -t treatGroup.BAM -c conGroup.BAM –GTF $species.gtf –TE $species.te –sortByPos -n TC –mode multi for all datasets.

### Differential expression analysis of TEs

In contrast to employing FPKM for coding gene normalization, we utilized reads per million mapped reads (RPM) to quantify the expression of TEs due to the potential variability in copy numbers and lengths among TEs. Subsequently, the RPM values for each sample underwent normalization using the R package DESeq2 (33). Differentially expressed TEs (DETEs) were identified using the DESeq algorithm, with criteria set as | log_2_ (fold change) | >1 and FDR <0.05.

### Quantification of identical loci from DETEs

We quantified uniquely mapped reads aligned to each DETE locus to discern specific TE loci expression levels, which may represent the primary rationale for the significant disparity between virus-infected and mock cells. TE loci overlapping with coding gene exons were excluded prior to quantification. Subsequently, we employed bedtools (37) to measure the expression of each DETE copy. The parameters for bedtools to quantify each DETE locus were as follows: for var in $(cat sample.txt); do bedtools intersect -a MER89-int.txt -b $var -c >./ERVCount_res/${var%-*}_MER89-int.txt; done.

### GREAT analysis

The majority of the coding genes in our genome were annotated with molecular functions, cellular components, and biological processes. However, non-coding regions lack such annotation, including non-coding RNA and repeated DNA regions. GREAT, which stands for “Genomic Regions Enrichment of Annotations Tool” (http://great.stanford.edu/public/html/index.php), facilitates functional enrichment analysis directly on the unannotated genomic regions (38). GREAT accomplishes this by assigning biological functions to non-coding genomic regions through the analysis of the functions of their neighboring coding genes. Therefore, we could leverage GREAT to investigate the cis functions of certain unannotated genomic regions, such as ERVs and TEs.

### Associating DETEs with their nearest genes

Given the potential relationship between TEs and their neighboring genes, we utilized the coding gene nearest to the DETEs to infer their functional implications across various species, employing the closest command of Bedtools (v2.27.1) (37). The parameters for determining the closest gene to TEs were bedtools closest -a genomic_TE.bed -b sorted_gencode.annotation.gtf | grep -o ‘gene_name\s”\w\+”‘ | uniq | cut -d ‘ ‘ -f 2 > ClosestAllLTR.txt.

### Cell culture and viral infection

Bone marrow-derived cells obtained from male C57BL/6J mice were stimulated with GM-CSF cytokines (ABclonal, RP01206) for 7 days at a final concentration of 40 ng/mL. Throughout the induction period, the cells were maintained in DMEM supplemented with 10% fetal bovine serum. The BMDMs were collected and seeded into a 12-well plate on the seventh day. Subsequently, the BMDM cells were infected with VSV (MOI of 0.1) and HSV-1 (F strain, MOI of 0.1) for 1 h. The media were aspirated after infection, and fresh DMEM was added (39). After 12 h, total RNA was extracted from the infected cells for downstream experiments.

### Quantitative RT-PCR (RT-qPCR)

RNA extraction from cells and tissues was conducted using the TRIZol reagent (TIANGEN). Subsequently, the purified RNA underwent reverse transcription using the HiScript II RT SuperMix (Vazyme, R223-01). The expression levels of target genes were assessed using SYBR Green qMix (Vazyme, Q311). The RT-qPCR data of this study represented the relative expression levels of target mRNAs normalized to the expression of *Gapdh*. The primers utilized in this investigation are listed in **Supplementary Table 8**.

### RNA-seq sample preparations

After 12 h of HSV-1 and VSV infection, total RNA was extracted from BMDMs using the TRIZol reagent (TIANGEN). Subsequently, the purified RNA samples from MOCK, HSV-1, and VSV infections were utilized to construct RNA libraries, which were then outsourced for sequencing by the GENEWIZ company (https://www.genewiz.com/).

### Statistical analysis

In this study, we employed the Python package SciPy (https://pypi.org/project/scipy/) for all statistical analyses, encompassing the one-way ANOVA test. The results were depicted as mean ± SEM. Statistical significance was determined with *p* values, where *p* values <0.05 were considered statistically significant (*), and *p* values <0.01, and *p* values < 0.001 were considered highly statistically significant (** and ***).

## Results

### Result 1: The transposable elements in the host genome were upregulated by SARS-CoV-2 infection

**Figure 1:**
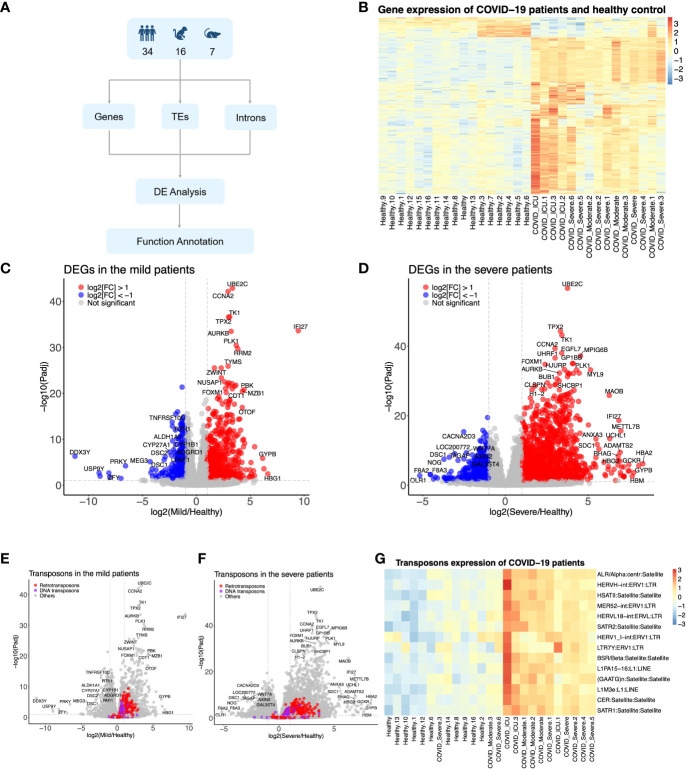
The SARS-CoV-2 infection upregulated the host genome’s transposable elements (TEs). **(A)** Diagram showing how to identify the TEs from humans, monkeys, and mice infected by SARS-CoV-2. **(B)** Heat map representing the expression pattern of COVID-19 patients. **(C, D)** Differentially expressed genes in the mild COVID-19 group and severe COVID-19 group, respectively. **(E, F)** Differentially expressed TEs in the mild COVID-19 group and severe COVID-19 group, respectively. **(G)** Heat map representing the expression pattern of part TEs from COVID-19 patients.

To explore the expression patterns and potential functions of TEs upon SARS-CoV-2 infection, we conducted a comprehensive and systematic analysis of RNA-seq data derived from hPBMCs and primary lung epithelial cells from monkeys and mice, both with and without SARS-CoV-2 infection (**Supplementary Table 1**). Utilizing a unified pipeline, as depicted in **Figure 1A**, we processed these datasets to identify and quantify the expression of coding genes and TEs, including ERVs, during SARS-CoV-2 infection. As illustrated in **Figure 1B**, SARS-CoV-2 infection significantly perturbed the expression of hundreds of coding genes and TEs in hPBMCs.

In comparison to the healthy control group, 833 genes exhibited upregulation, while 368 genes demonstrated downregulation in the mild COVID-19 group (**Figure 1C**). These genes were found to be enriched in signaling pathways such as neutrophil extracellular trap formation, systemic lupus erythematosus, virus carcinogenesis, and the p53 signaling pathway, among others (**Supplementary Figure 1A**). In contrast, the combined severe group displayed 2,048 upregulated and 675 downregulated elements (**Figure 1D**) compared to the control group, which were similarly enriched in pathways like neutrophil extracellular trap formation, systemic lupus erythematosus, and other pathways such as the calcium signaling pathway and focal adhesion (**Supplementary Figure 1B**). Certain TEs exhibited significant upregulation in both the mild and severe groups (**Figure 1G**)—for instance, 135 DNA transposons and 489 retrotransposons in the mild group and 124 DNA transposons and 540 retrotransposons in the severe group were upregulated, respectively (**Figures 1E, F**). However, no downregulated TEs met the criteria (log_2_(FC) < -1X and FDR <0.05). The annotations of DEGs in datasets from monkeys and mice were consistent with those in humans, including pathways such as the NF-kappa B signaling pathway, PI3K-Akt signaling pathway, TNF signaling pathway, inflammatory response, immune system response, response to lipopolysaccharide (LPS), response to virus, etc. (**Supplementary Figures 1E, F**). Moreover, the number of TEs was increased in monkeys and mice upon SARS-CoV-2 infection (**Supplementary Figures 1C, D**). In conclusion, these findings suggested that TE subfamilies were induced by SARS-CoV-2 infection across humans, monkeys, and mice.

### Result 2: Characterization of upregulated endogenous retrovirus after SARS-CoV-2 infection

**Figure 2:**
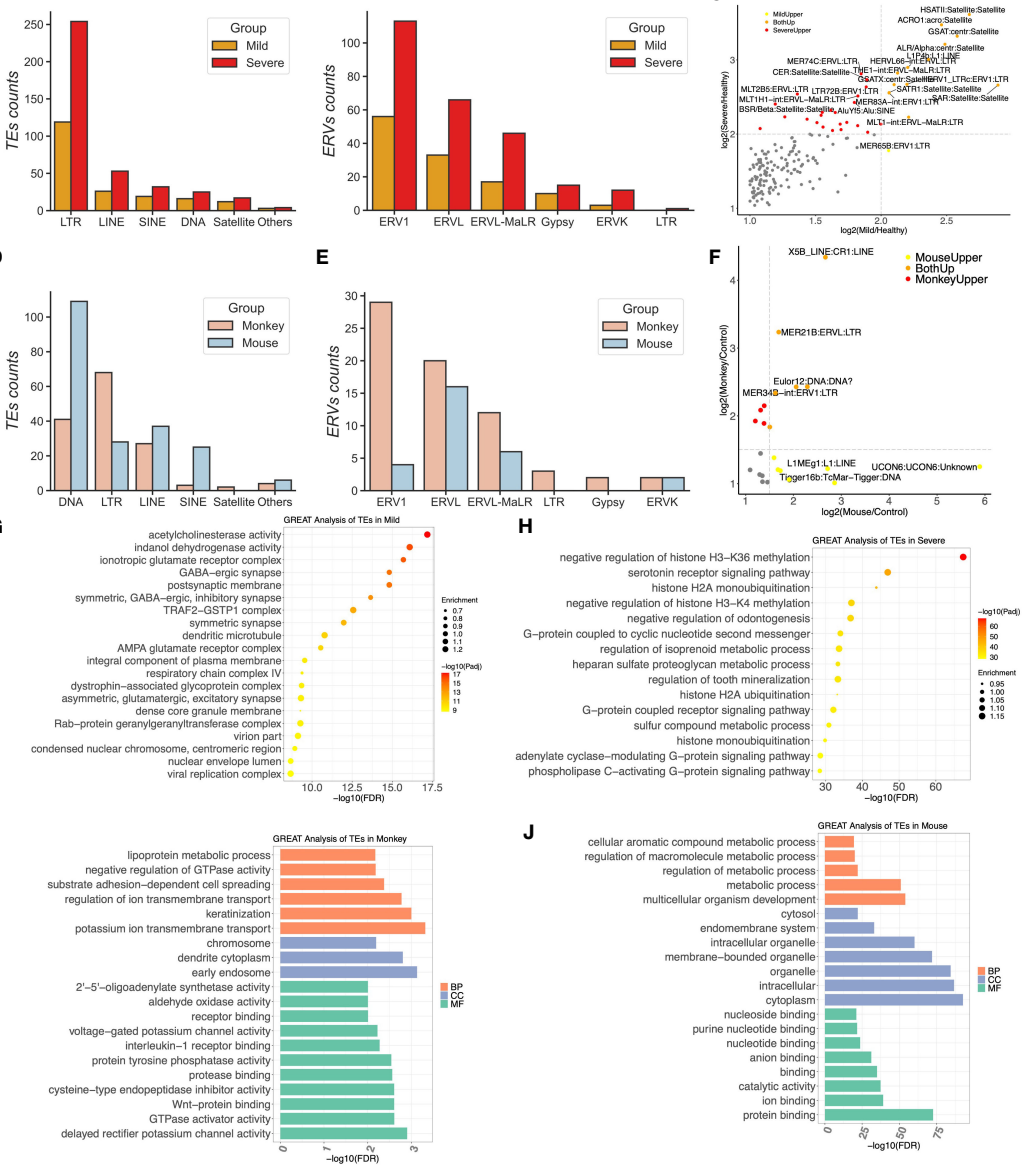
The most upregulated transposable elements (TEs) were subfamilies of endogenous retroviruses in COVID-19 patients. **(A)** Compositions of upregulated TEs in COVID-19 patients. **(B)** Distributions of long terminal repeats (LTRs) in the mild and severe groups. **(C)** The scatter plotting represents the correlation of TEs between the mild and severe patients. **(D)** Compositions of upregulated TEs in the monkeys and mice infected by SARS-CoV-2. **(E)** Distributions of LTRs in the monkeys and the mice group, respectively. **(F)** The scatter plotting represents the correlation of TEs between the mice and monkeys. **(G, H)** GREAT prediction analysis of upregulated TEs from the mild and severe patients, respectively. **(I, J)** GREAT prediction analysis of upregulated TEs from the monkeys and mice, respectively.

Utilizing the TEcount tool with the standard protocol as previously outlined, we identified several TE subfamilies expressed across different species infected by SARS-CoV-2 (**Supplementary Table 3**). The ERV subfamilies exhibited significant upregulation in humans and monkeys following SARS-CoV-2 infection, albeit not in transgenic mice with the hACE2 receptor. In COVID-19 patients, the mild group displayed 195 significantly upregulated TEs identified by DESeq2 compared to the healthy control group. These included 119 LTR ERV members, 26 LINE members, 19 SINE members, 16 DNA repeat members, and 12 Satellite members (**Figure 2A**). Meanwhile, the severe group exhibited 385 upregulated TEs compared to the healthy group, comprising 254 LTR ERVs, 53 LINE members, 32 SINE members, 25 DNA members, 17 Satellite members, and others (**Figure 2A**). Notably, the top upregulated TE subfamilies in both the mild and severe conditions were LTR ERVs, encompassing the ERV1, ERVL, ERVL-MaLR, Gypsy, and ERVK subfamilies. Specifically, the ERV elements upregulated in the mild group included 56 ERV1 members, 33 ERVL members, 17 ERVL-MaLR members, 10 Gypsy members, and three ERVK members (**Figure 2B**). Similarly, the severe group exhibited 133 ERV1, 66 ERVL, 46 ERVL-MaLR, 15 Gypsy, and 12 ERVK members in the upregulated ERV family compared to the healthy control group (**Figure 2B**)—for instance, HERV1_I-int and HERV9-int emerged as the top two upregulated TEs in the severe group but not in the mild group, both belonging to the ERV1 subfamily (**Supplementary Table 3**), suggesting the potential significance of the ERV1 subfamily in COVID-19 development. Among these TEs, HSATII, ACRO1, SATR1, and SAR from the Satellite family exhibited upregulation in both the mild and severe groups (**Figure 2C**).

In the sample of monkeys infected by SARS-CoV-2, 145 TEs were detected, comprising 68 LTR ERVs, 41 DNA repeats, 27 LINE, 3 SINE, and two Satellite elements (**Figure 2D**). Among these, 29 ERV1 members, 20 ERVL members, 12 ERVL-MaLR members, two ERVK members, and two Gypsy members constituted the upregulated ERVs in the lung tissues of the monkeys (**Figure 2E**). However, in transgenic mice expressing ACE2, the top upregulated TE subfamilies were from the DNA repeat subfamily rather than the LTR ERV subfamily (**Figure 2D**), with 109 members identified, including only 28 upregulated LTR ERV members. The ERV subfamily of mice comprised 16 ERVL members, six ERVL-MaLR members, four ERV1 members, and two ERVK members (**Figure 2E**). Notably, X5B_LINE : CR1:LINE, MER21B:ERVL : LTR, and MER34B-int:ERV1:LTR were upregulated in both the monkey and mouse groups, while L1MEg1:L1:LINE and Tigger16b:TcMar-Tigger : DNA were specifically upregulated in the mouse group (**Figure 2F**).

To assess the functions of these upregulated TEs within host cells, we conducted GREAT analysis on the ERV loci of humans that exhibited a significant upregulation compared to the healthy control group upon infection. Remarkably, most of these upregulated ERVs in severe COVID-19 patients were located close to genes involved in the negative regulation of histone H3-K36 methylation, negative regulation of histone H3-K4 methylation, G-protein coupled receptor signaling pathway, G-protein associated with cyclic nucleotide second messenger, histone H2A ubiquitination, and ER ubiquitin ligase complex. Conversely, the upregulated ERVs in the mild group were enriched in the virion part, viral replication complex, respiratory chain complex IV, and TRAF2–GSTP1 complex (**Figures 2G, H** and **Supplementary Table 4**).

We applied the same pipeline to analyze 28 upregulated ERV members in the hACE2 mouse, which were also enriched in gene expression, metabolic processes, catalytic activity, protein binding, ion binding, etc. (**Figure 2J**). However, the GREAT website only supports genome region enrichment annotation for human, mouse, and zebrafish species. Thus, we first identified the genes closest to these 68 upregulated ERV1 members in the monkey genome to obtain the DE nearest genes (DENGs) using the bedtools closest subcommand. Subsequently, we used these DENGs to conduct the GO annotation using DAVID instead of the GREAT database. The GO enrichment analysis of these ERVs included DNA replication origin binding, RNA polymerase II sequence-specific DNA binding transcription factor binding, poly(A) binding, receptor complex, receptor binding, early endosome, and others, suggesting that these DENGs may be involved in DNA replication and signaling transduction in host cells (**Figures 2I, J**).

### Result 3: ERV1 subfamily may contribute to the severity of COVID-19

**Figure 3:**
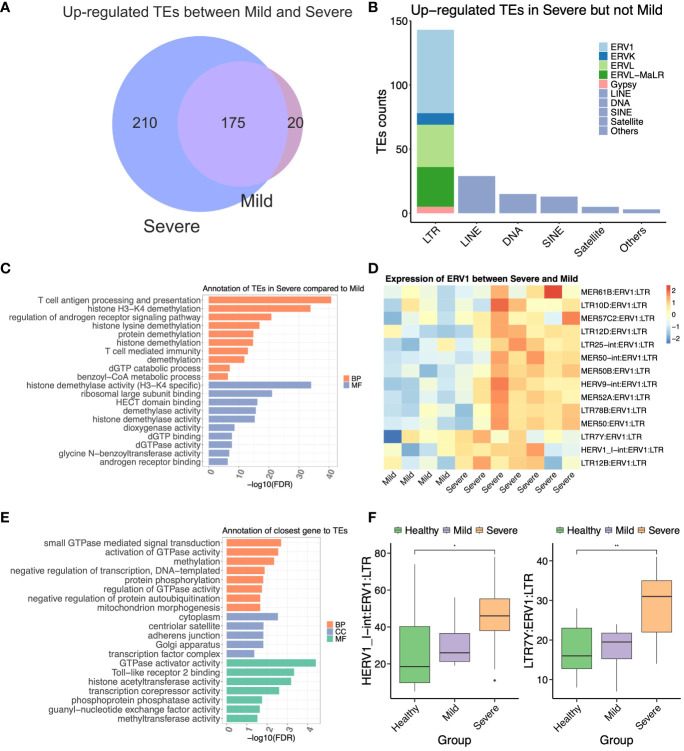
The ERV1 subfamily located near antiviral response genes may contribute to the severity of COVID-19. **(A)** Venn diagram representing the upregulated transposable elements (TEs) between mild and severe COVID-19 patients. **(B)** Compositions of the differentially expressed TEs (DETEs) between mild and severe patients. **(C)** GREAT prediction analysis of upregulated TEs in the severe group compared to the mild group. **(D)** Heat map of differentially expressed ERV1 subfamily between mild and severe patients. **(E)** Gene Ontology annotation of the differentially expressed nearest genes of the ERV1 subfamily between mild and severe patients. **(F)** The boxplot represents the expression of two ERV1 members from COVID-19 patients. The statistical significance was determined by one-way ANOVA with Bonferroni’s multiple-comparisons tests **(F)**. *P < 0.05, **P < 0.01.

Next, we aimed to differentiate the upregulated TEs between the severe and mild groups, which could shed light on potential factors contributing to the development of severe COVID-19. We observed that 210 TEs were exclusively upregulated in the severe group, but not in the mild group (**Figure 3A**). These upregulated TEs in the severe group comprised 145 LTR elements, 29 LINE elements, 15 DNA repeat elements, 13 SINE elements, five Satellite elements, and others (**Figure 3B**). Among the LTR elements, there were 65 ERV1 members, 33 ERVL members, 31 ERVL-MaLR members, nine ERVK members, and five Gypsy members, among others (**Figure 3B**), suggesting that the ERV1 subfamily may play crucial roles in the pathogenesis of COVID-19.

To further elucidate the distinctions between the severe and mild groups, we conducted GREAT analysis on all 145 differentially expressed LTR elements specifically upregulated in the severe group. Remarkably, most of these LTRs were enriched in pathways related to T cell antigen processing and presentation, histone H3-K4 demethylation, histone lysine demethylation, T cell-mediated immunity, dGTP binding, dGTPase activity, etc. (**Figure 3C**). Subsequently, we focused on the top 15 upregulated ERV1 members of the LTR family in the severe group compared to the mild group (**Figure 3D**). To assess the potential role of the upregulated ERV1 subfamily, we identified 332 DENGs associated with these 15 DE ERV1 members, totaling 3,450 copy numbers. The GO annotation of these DENGs revealed functions such as small GTPase-mediated signal transduction, activation of GTPase activity, Toll-like receptor 2 binding, transcription corepressor activity, histone acetyltransferase activity, and methyltransferase activity, among others (**Figure 3E**). Additionally, we observed that genes related to Toll-like receptor 2 binding, histone acetyltransferase activity, and transcription corepressor activity were located in proximity to HERV1_I-int:ERV1:LTR and LTR7Y:ERV1:LTR. Notably, these two ERV1 members were significantly upregulated in the severe but not in the moderate groups (**Figure 3F**), suggesting their potential as biomarkers for predicting severe COVID-19 patients. These findings suggested that certain upregulated ERV1 subfamily members likely interacted with genes related to the immunity and epigenetic modification of the host, potentially contributing to the pathogenesis of COVID-19.

### Result 4: Validation of the robustness of this pipeline using BMDMs infected by VSV and HSV-1

**Figure 4:**
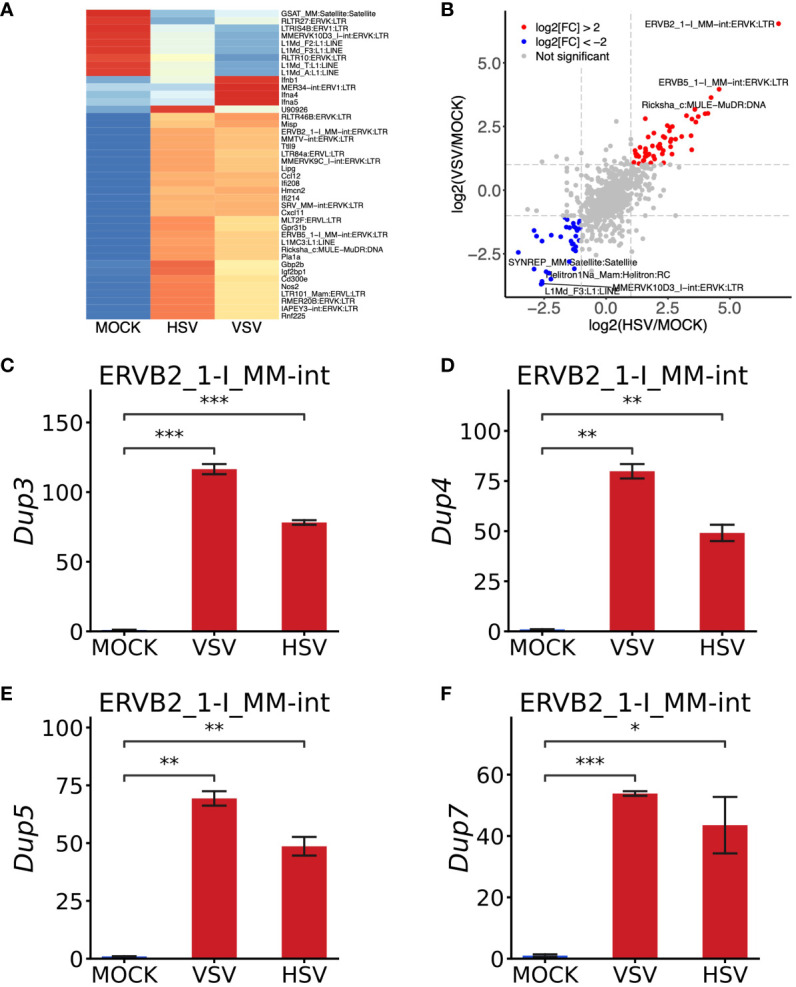
Validation of the robustness of this pipeline using bone marrow-derived macrophages (BMDMs) infected by VSV and HSV-1. **(A)** Heat map of interferon‐related genes and TEs in BMDMs upon VSV and HSV-1 treatment. **(B)** Scatterplot representing VSV/MOCK and HSV-1/MOCK group correlations. Compared to the MOCK group, the fold change was transformed by “-log_2_”. The transposable elements were upregulated by VSV and HSV-1, shown as red dots, while those blue dots represent those that were downregulated by two treatments. **(C–F)** RT-qPCR analysis of four copies from ERVB2_1-I_MM-int:ERVK: LTR. RT-qPCR data are shown from three independent experiments. The error bar represents means ± SEM. Statistical significance was determined by one-way ANOVA with Bonferroni’s multiple-comparisons tests **(C–F)**. *P < 0.05, **P < 0.01, ***P < 0.001.

In another independent experiment to demonstrate our pipeline’s robustness in predicting TEs and ERVs, we initially isolated BMDMs from C57BL/6J mice. Following induction, BMDMs were treated with VSV and HSV-1 for 12 h, after which total RNA was extracted for bulk RNA-seq analysis. Employing the unified pipeline described earlier, we identified a total of 1,082 TEs induced upon VSV and HSV-1 treatment (**Supplementary Table 5**). A portion of the DEGs and DETEs, respectively, are depicted in **Figure 4A**. The top three upregulated TE members included ERVB2_1-I_MM-int:ERVK: LTR, ERVB5_1-I_MM-int:ERVK : LTR, and Ricksha_c : MULE-MuDR : DNA. Conversely, MMERVK10D3_I-int:ERVK : LTR, Helitron1Na_Mam : Helitron:RC, L1Md_F3:L1:LINE, and SYNREP_MM : Satellite:Satellite emerged as the top four downregulated members following both VSV and HSV-1 treatments compared to the mock group (**Figure 4B**).

Subsequently, we directed our attention to the most upregulated member, ERVB2_1-I_MM-int, which belongs to the ERVK sub-family and LTR family. According to the TE annotation file of the mouse (mm10_rmsk_TE.gtf), ERVB2_1-I_MM-int was represented by 83 copies in the mouse genome (**Supplementary Table 6**). Using Bedtools, we meticulously assessed the expression of each copy. It emerged that ERVB2_1-I_MM-int_dup_3/_4/_5/_7 were the primary contributors to the upregulation of ERVB2_1-I_MM-int (**Supplementary Table 6**). Subsequently, we retrieved the DNA sequence of these four copies and designed primers for RT-qPCR analysis. Remarkably, all of these specific copies exhibited significant induction (**Figures 4C–F**), consistent with the prediction of ERVB2_1-I_MM-int from RNA-seq data, thus validating the accuracy and reliability of the pipeline used to identify TEs and ERVs.

Interestingly, these four copies of ERVB2_1-I_MM-int were found in close proximity to *Ifi208*, *Ifi213*, *Mlph*, and *Serpinb10* in the mouse genome (**Supplementary Table 7**). Notably, *Ifi208* and *Ifi213* are two interferon-stimulated genes (ISGs) that play essential roles in anti-viral immunity (40, 41). Upon treatment with VSV and HSV-1, both *Ifi208* and *Ifi213* were significantly upregulated, indicating a potential interaction between ERVB2_1-I_MM-int and *Ifi208* as well as *Ifi213*. *Serpinb10*, on the other hand, is a member of the serpin peptidase inhibitor family and is involved in the apoptosis process induced by TNF (42). Taken together, these results suggested that the upregulated ERVs may interact with some ISGs and be involved in the host’s innate immune responses.

## Discussion

Indeed there has been a noticeable increase in studies leveraging NGS technology to explore the gene expression patterns of host cells during viral infections, which has significantly contributed to our understanding of virus–host immune system interactions. However, many of these studies have primarily focused on investigating the expression profiles of coding genes, often neglecting the expressions of TEs. TEs encompass diverse families, such as ERVs, which belong to the LTR family, LINEs family, SINE family, DNA transposons, etc. These elements have played crucial roles in shaping our genome in over millions of years of evolution (12, 23, 43, 44). The emergence of COVID-19 has posed a substantial threat to global health (45). Increasing evidence suggests a potential correlation between the pathology of SARS-CoV-2 and the activation of ERVs—for instance, Balestrieri et al. observed an association between the expression of the HERV-W envelope in T lymphocytes and the respiratory outcome of COVID-19 patients (26), a finding supported by the work of Garcia-Montojo and Giménez-Orenga (24, 25). Additionally, the expression of HERV-K in the respiratory tract has been linked to the physiopathology of COVID-19 (46). Moreover, HERV-K113-ENV has been proposed as a biomarker to assess the severity of SARS-CoV-2 infection (29, 46). Previous studies have also suggested that HERV-K (HML-2) may stimulate interferon production in COVID-19 patients (47). A recent survey of HERVs has indicated that HERVs and inflammatory mediators detected in nasal mucosa could serve as predictive biomarkers of COVID-19 (48). While Nicole et al. demonstrated the dynamic modulation of the HERV transcriptome across COVID-19 convalescent stages and in individuals retesting positive after convalescence (30), the comparison of TEs and ERVs among healthy donors and individuals with mild and severe stages of COVID-19, as well as across species such as monkeys and mice infected by SARS-CoV-2, warrants further exploration.

This study employed comprehensive analysis methods and tools to investigate the expression patterns and potential functions of TEs using publicly available RNA-seq data of SARS-CoV-2 infection. The findings revealed that SARS-CoV-2 infection could upregulate certain TEs, including ERV subfamilies, across human, monkey, and mouse models. Moreover, the GREAT prediction analysis highlighted that genomic regions associated with these upregulated TEs in COVID-19 patients might be involved in the negative regulation of histone H3-K36 methylation, histone H3-K4 methylation, G-protein coupled receptor signaling pathway, histone H2A ubiquitination, ER ubiquitin ligase complex, the virion part, viral replication complex, and respiratory chain complex IV. Remarkably, 210 ERVs were identified as upregulated explicitly in severe COVID-19 cases. Genes proximal to these differentially expressed ERVs were enriched in functions such as Toll-like receptor 2 binding, T cell activation, and histone H3-K4 demethylation process. Notably, HERV1_I-int: ERV1:LTR and LTR7Y: ERV1:LTR emerged as potential biomarkers for predicting COVID-19 severity. Additionally, a re-analysis of 24 RNA-seq samples from primary human bronchial epithelial cells (HBECs), both infected and uninfected with SARS-CoV-2 for 24 h (GSE147507), was conducted (**Supplementary Table 9**). The analysis indicated fewer upregulated TEs in HBECs infected by SARS-CoV-2 compared to hPBMCs from COVID-19 patients. Furthermore, the top five upregulated TEs in these epithelial cells infected by SARS-CoV-2 were identified as LTR81A:Gypsy : LTR, LTR35B:ERV1:LTR, LTR85b:Gypsy : LTR, LTR75:ERVL : LTR, and MER67C:ERV1:LTR (**Supplementary Table 10**), diverging from the findings of hPBMCs. To confirm the findings obtained from hPBMCs, we utilized two additional GEO datasets (GSE206263 and CRA002390), which showed a substantial overlap of differentially expressed TEs and ERVs with the initial hPBMCs analysis (**Supplementary Table 14**). The two potential ERV markers exhibited a significant upregulation in the severe group compared to mild COVID-19 patients (GSE206263). Furthermore, RT-qPCR was utilized to validate the expression of some upregulated ERVs, thereby confirming the predictions using BMDMs from mice infected by HSV-1 and VSV. Indeed in our supplementary experiment, we treated BMDMs with VSV (MOI of 0.1) for 24 h. As expected, the results of VSV infection for 24 h were largely consistent with those of 12 h of VSV infection, as illustrated in **Supplementary Table 11**—for instance, ERVB2_1-I_MM-int:ERVK : LTR and ERVB5_1-I_MM-int:ERVK : LTR were significantly upregulated at both 12 and 24 h post-VSV infection. Specifically, the expression of these ERVs was higher at 12 h, indicating that the expression pattern of ERVs may represent an early event in viral infection. To assess specificity to viral stress, RAW 264.7 cells were treated with LPS for 24 h (**Supplementary Table 12**), and publicly available RNA-seq data from human dermal fibroblasts treated with poly(I:C) for 72 h (GSE223543) were re-analyzed. Few ERVs exhibited upregulation upon LPS and poly(I:C) treatment (**Supplementary Table 13**), distinct from VSV and HSV infection. Collectively, these findings underscored the potential importance of TEs and ERVs in SARS-CoV-2 infection, highlighting their utility as diagnostic biomarkers and therapeutic targets.However, further wet experiments are needed to demonstrate whether the pattern recognition receptors of host cells such as RIG-I, MDA5, or cGAS can recognize the products of these ERVs, such as ssRNA, dsRNA, or dsDNA (49–52). Additionally, this study did not investigate the transposed activity of these most upregulated ERVs directly upon SARS-CoV-2 infection—for instance, it remains unclear whether these ERVs produce all the necessary proteins to form complete TEs capable of transposition in the genome or whether these ERVs can transcribe the RNA of certain viruses and reinfect the host. These are important questions that warrant investigation in future studies. In conclusion, this study provides valuable evidence supporting the notion that SARS-CoV-2 can upregulate ERVs, which may be implicated in the pathogenesis of COVID-19. Furthermore, these results suggest that the upregulated ERVs, including TEs, may be involved in the host’s immune responses under viral stress. Continued research in this area will enhance our understanding of the intricate interplay between viral infections and host genomic elements.

## Data availability statement

The datasets presented in this study can be found in online repositories. The names of the repository/repositories and accession number(s) can be found below: GSE244322 (GEO).

## Ethics statement

Ethical approval was not required for the studies on humans in accordance with the local legislation and institutional requirements because only commercially available established cell lines were used. The animal study was approved by Institutional Animal Care and Use Committee. The study was conducted in accordance with the local legislation and institutional requirements.

## Author contributions

XG: Software, Supervision, Visualization, Writing – original draft, Writing – review & editing. YZ: Validation, Writing – review & editing. FY: Writing – review & editing.

## Supplementary material

Supplementary Figure 1 Transcriptome dynamics induced by SARS-CoV-2 infection in different host cells. **(A**, **B)** KEGG enrichment analysis of differentially expressed genes (DEGs) in the mild COVID-19 group and severe COVID-19 group, respectively. **(C**, **D)** Heat map representing the expression pattern of part transposable elements from the transgenetic mice with hACE2 receptor and monkeys (hACE2 mice), respectively. **(E**, **F)** KEGG and GO enrichment analysis of DEGs from the hACE2 mice, respectively.

Supplementary Table 1 Meta-information of the 57 RNA-seq samples from humans, monkeys, and mice infected by SARS-CoV-2.

Supplementary Table 2 Group information of the COVID-19 patients in our analysis.

Supplementary Table 3 Expression matrix of coding genes and transposable elements from COVID-19 patients.

Supplementary Table 4 Annotation of upregulated transposable elements from COVID-19 patients using GREAT analysis.

Supplementary Table 5 Expression matrix of coding genes and transposable elements from mouse bone marrow-derived macrophages infected by VSV and HSV-1.

Supplementary Table 6 Distributions and expression count of ERVB2_1-I_MM-int:ERVK: LTR.

Supplementary Table 7 Closest genes of ERVB2_1-I_MM-int:ERVK: LTR.

Supplementary Table 8 Primer sequences used in this study.

Supplementary Table 9 Expression matrix of coding genes and transposable elements from primary human bronchial epithelial cells with or without SARS-CoV-2 infection (GSE147507).

Supplementary Table 10 Differentially expressed analysis of coding genes and transposable elements from primary human bronchial epithelial cells with or without SARS-CoV-2 infection.

Supplementary Table 11 Expression matrix of coding genes and transposable elements from mouse bone marrow-derived macrophages infected by VSV for 24 h.

Supplementary Table 12 Expression matrix of coding genes and transposable elements from RAW 264.7 cells upon lipopolysaccharide treatment for 24 h.

Supplementary Table 13 Expression matrix of coding genes and transposable elements from human dermal fibroblasts treated with poly(I:C) for 72 h (GSE223543).

Supplementary Table 14 Differential expression analysis of coding genes and transposable elements of two independent datasets for validation (GSE206263 and CRA002390).
