# Supplementary figures and images for: Identification and characterization of endogenous retroviruses upon SARS-CoV-2 infection
Source: Front Immunol. 2024 Apr 5;15:1294020. doi: 10.3389/fimmu.2024.1294020 (PMC11026653; doi:10.3389/fimmu.2024.1294020)

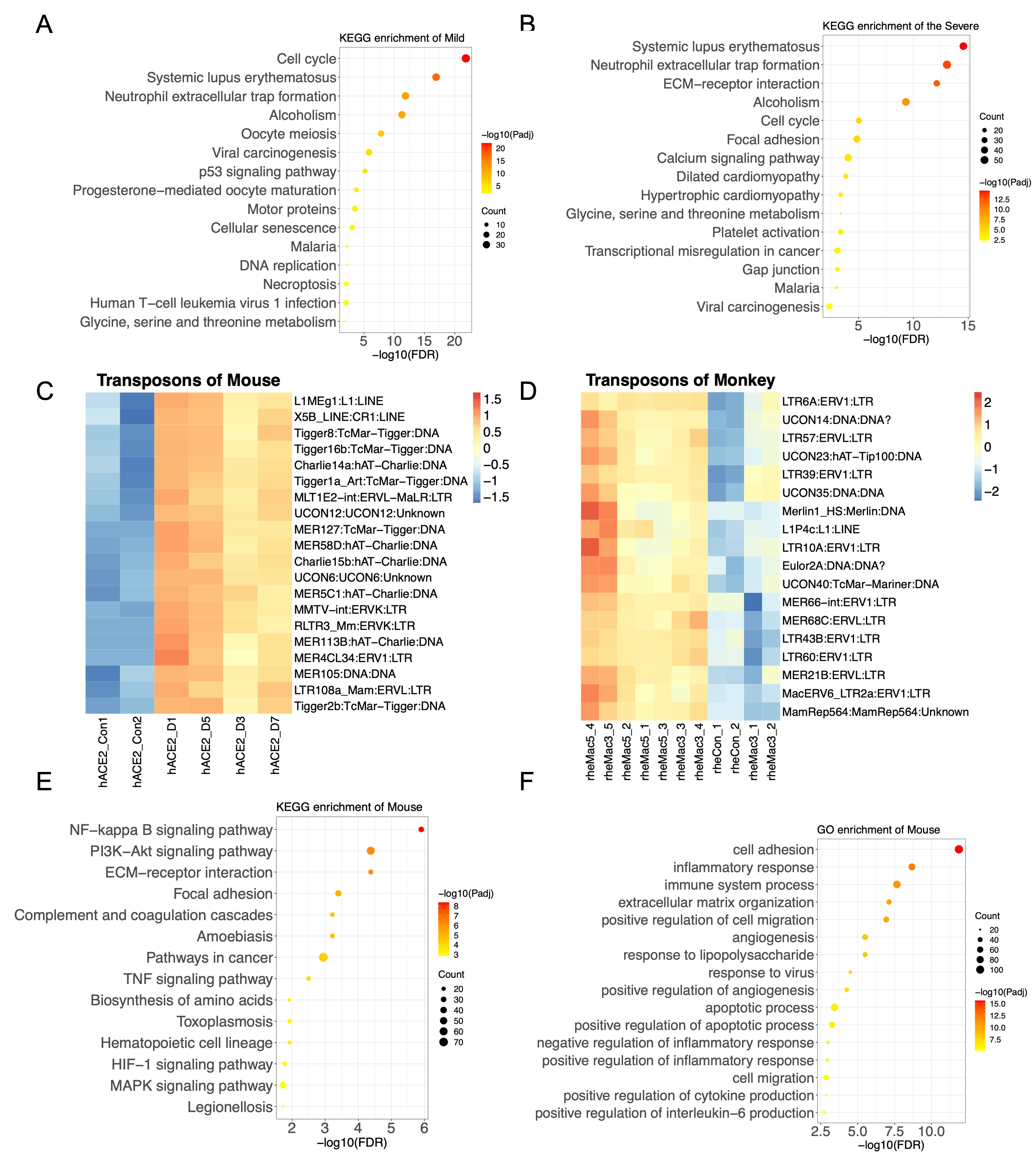

Supplement: Supplementary Figure 1 — Transcriptome dynamics induced by SARS-CoV-2 infection in different host cells. (A, B) KEGG enrichment analysis of differentially expressed genes (DEGs) in the mild COVID-19 group and severe COVID-19 group, respectively. (C, D) Heat map representing the expression pattern of part transposable elements from the transgenetic mice with hACE2 receptor and monkeys (hACE2 mice), respectively. (E, F) KEGG and GO enrichment analysis of DEGs from the hACE2 mice, respectively. [file Image_1.jpeg]
